# Changes in intraocular pressure values measured with noncontact tonometer (NCT), ocular response analyzer (ORA) and corvis scheimpflug technology tonometer (CST) in the early phase after small incision lenticule extraction (SMILE)

**DOI:** 10.1186/s12886-016-0381-3

**Published:** 2016-11-18

**Authors:** Yang Shen, Xiangjian Su, Xiu Liu, Huamao Miao, Xuejun Fang, Xingtao Zhou

**Affiliations:** 1 Key Lab of Myopia, Ministry of Health, P.R. China, 19 Baoqing Road, Shanghai, 200031 China; 2 Department of Ophthalmology, EYE & ENT Hospital of Fudan University, Shanghai, China, 83 Fenyang Road, Shanghai, 200031 China; 3 Shenyang Aier Eye Hospital, 11 Shiyiwei Road, Shenyang, Liaoning Province 110000 China

**Keywords:** Intraocular pressure, Corneal biomechanical property, Corneal deformation parameter, Small incision lenticule extraction, Ocular response analyzer, Corvis scheimpflug technology tonometer

## Abstract

**Background:**

Corneal biomechanical properties are always compromised after corneal refractive surgeries thus leading to underestimated intraocular pressure (IOP) that complicates the management of IOP. We investigated the changes in postoperative baseline of IOP values measured with noncontact tonometer (NCT), ocular response analyzer (ORA) and corvis scheimpflug technology (CST) in the early phase after small incision lenticule extraction (SMILE).

**Methods:**

Twenty-two eyes (−6.76 ± 1.39D) of 22 moderate and high myopes, (28.36 ± 7.14 years, 12 male and 10 female) were involved in this prospective study. IOP values were measured using a non-contact tomometer (NCT-IOP), an ocular response analyzer (corneal-compensated IOP, IOPcc and Goldmann-correlated IOP, IOPg) and a Corvis scheimpflug technology tonometer (CST-IOP) preoperatively, at 20 min and 24 h, postoperatively. Repeated measures analysis of variance (RM-ANOVA), Pearson’s correlation analysis and multiple linear regression models (stepwise) were performed. Cut-off *P* values were 0.05.

**Results:**

Except for IOPcc, NCT-IOP, IOPg, and CST-IOP values significantly decreased after SMILE procedure (All *P* values <0.05). ΔCCT, as well as ΔMRSE and ΔKm, did not significantly correlated with ΔNCT-IOP, ΔIOPcc, ΔIOPg or ΔCST-IOP, (all *P* values >0.05). Multiple linear regression models (stepwise) showed that the practical post-operative IOP value was the main predictor of the theoretical post-operative NCT-IOP, IOPcc and IOPg values (all *P* values <0.001). The postoperative applanation time 1 (AT1) value (B = 8.079, t = 4.866, *P* < 0.001), preoperative central corneal thickness (CCT) value (B = 0.035, t = 2.732, *P* = 0.014) and postoperative peak distance (PD) value (B = 0.515, t = 2.176, *P* = 0.043) were the main predictors of the theoretical post-operative CST-IOP value.

**Conclusions:**

IOP values are underestimated when assessed after SMILE by using NCT-IOP, IOPg and CST-IOP. The practical postoperative IOPcc value and theoretical post-operative CST-IOP value may be more preferable for IOP assessment in the early phase after SMILE.

**Trial registration:**

Current Controlled Trials ChiCTRONRC13003114. Retrospectively registered 17 March 2013

## Background

With the booming popularity of refractive surgeries, nowadays, intraocular pressure (IOP) management is not only essential for patients with glaucoma but also for those myopes who underwent refractive surgeries as long-term use of topical steroid may cause steroid-induced ocular hypertension, and primary open-angle glaucoma is a common complication accompanying myopia [1, 2]. However, corneal refractive surgeries remove corneal tissue, modify corneal shape and compromise corneal biomechanical properties thus leading to underestimated IOP values [3] and obscure the diagnosis of ocular hypertension. Noncontact tonometer (NCT), ocular response analyzer (ORA) and corvis scheimpflug technology tonometer (CST) are three most commonly employed instruments for clinical IOP assessment. Goldmann applanation tonometer is the gold standard method for IOP assessment; nonetheless, its accuracy still depends on central corneal thickness (CCT), anterior corneal curvature and other potential factors that may affect corneal biomechanical properties [4–6]. The permanent corneal flap and the incomplete Bowman’s layer, caused by laser-assisted in situ keratomileusis (LASIK) or surface ablation techniques, are another two major factors that weaken corneal stiffness and affect postoperative IOP assessment [7, 8].

Previously, large quantities of formulas have been raised to correct IOP values following corneal refractive surgeries [9–11]. However, various confounding factors including surgical designs (i.e., flap thickness, residual stromal bed thickness, optic zone diameter and ablation depth), individual differences (i.e., age, gender, race, refractions, corneal curvature corneal hydration and post-operative wound healing response) [12] and long-term postoperative topical steroid usage make these formulas widely divergent.

Femtosecond laser small incision lenticule extraction (SMILE) is a flapless and minimally invasive corneal refractive surgery [3, 13]. With a refractive stromal lenticule extracted from a 2 mm-long side-cut, the integrity of corneal structure (including the Bowman’s layer) and corneal biomechanical properties are maximally maintained [14]. Technically, SMILE procedure only modifies corneal curvature and corneal thickness.

To minimize the interference of those confounding factors, in the present study, IOP values were obtained preoperatively, at 20 min postoperatively and at 24 h post-operatively by using the three frequently employed noncontact tonometers. We hypothesis the theoretical post-operative IOP values should be similar with the preoperative values. The gap between preoperative and postoperative IOP values should be dominantly caused by the surgery itself. As IOP values are always underestimated following corneal refractive surgeries [15], to investigate and establish a statistical model for compensating the gap between the pre-operative and the postoperative IOP values by involving corneal biomechanical parameters should be meaningful for clinical IOP management.

## Methods

This prospective study was registered in Chinese Clinical Trial Registry (Trial registration: Current Controlled Trials ChiCTRONRC13003114. Retrospectively registered 17 March 2013), approved by ethics committee of Eye and ENT Hospital, Fudan University and was conducted with due regard to the tenets of the Declaration of Helsinki. Written informed consent was obtained from the participants after explanation of procedure was given.

## Participants

Twenty-two moderate and high myopes (28.4 ± 7.1 years, 12 male and 10 female) were recruited in this prospective study at the Department of Ophthalmology, Eye and ENT Hospital, Fudan University. All the right eyes (−6.76 ± 1.39D) were analyzed.

### Pre-operative examinations

Each participant underwent routine preoperative ophthalmologic examinations, including uncorrected distance visual acuity (UDVA), manifest refraction, best-corrected distance visual acuity (BDVA), slit lamp examination and fundus examination. Corneal topography was measured with a three-dimensional anterior segment analyzer (Pentacam HR, Typ70900, Oculus Optikgeräte GmbH, Wetzlar, Germany). The mean anterior corneal curvature (Km) and CCT were recorded. Preoperative IOP was measured with a noncontact tonometer (NCT, TX-20 Full Auto Tonometers, Canon, Japan), an ocular response analyzer (ORA, Reichert Inc, Depew, New York, USA) and a corvis scheimpflug technology tonometer (CST, Oculus Optikgeräte GmbH, Wetzlar, Germany). The sequence of measurement using these three instruments was arranged randomly. All the IOP measurements were obtained in a sitting position. NCT continuously obtained three valid readings and the mean value was calculated automatically. ORA provided values of corneal-compensated IOP (IOPcc), Goldmann-correlated IOP (IOPg), corneal hysteresis (CH) and corneal resistance factor (CRF). Four measurements were obtained in each eye and the one with the highest waveform score (WS) was recorded. CST-IOP and corneal deformation parameters were measured using CST. The measurement with an “OK” in Quality Specification (QS) was recorded [13].

### Surgical technique

SMILE procedures were performed under topical anaesthesia using three drops of 0.4% Oxybuprocaine Hydrochloride (Santen Pharmaceutical Co., Ltd., Japan). One surgeon (ZXT) performed all the procedures with the VisuMax femtosecond laser system (Carl Zeiss Meditec AG, Germany). The intended thickness of the upper tissue arcade was set 120 μm, and its diameter was 7.5 mm. The diameter of the lenticule was 6.7 mm. The side cuts were set 90° apart at a width of 2 mm. The refractive lenticule of the intra-stromal corneal tissue was extracted through the side-cut opening using a modified serrated McPherson forceps (Geuder, GmbH, Heidelberg, Germany) [3].

### Post-operative examinations and topical eye drops usage

UDVA, manifest refraction, BDVA, slit lamp examination, NCT-IOP, IOPcc, IOPg, CST-IOP were measured again at postoperative 20 min and postoperative 24 h. Corneal topography was measured again at 24 h postoperatively. Topical steroid (fluorometholone 0.1%; Santen Pharmaceutical Co., Ltd.), topical antibiotics (ofloxacin ophthalmic solutions 0.5%; Santen Pharmaceutical Co., Ltd.) and artificial tear (hypromellose 2910, dextran 70, glycerol eye drops; Alcon Laboratories, Inc., Fort Worth, TX) were used at 3-h intervals after SMILE procedure. At the first day post-operatively, topical steroid, topical antibiotics and artificial tear were employed for 4 times per day [13].

### Data analysis and statistical evaluation

Statistical analysis was performed using SPSS 19 (SPSS Inc., IBM, USA). All the data were tested for normality using the Kolmogorov-Smirnov test. Repeated measures analysis of variance (RM-ANOVA) with LSD post hoc comparisons was performed to evaluate the changes in NCT-IOP, IOPcc, IOPg and CST-IOP over time. Pearson’s correlation analysis was applied to detect the potential correlations between these variables. Stepwise multiple linear regression model analysis was performed to predict theoretical post-operative IOP values. To minimize the effect of corneal epithelium edema, the IOP value obtained at 20 min post-operatively was excluded from the analysis. Cut-off P values were 0.05.

## Results

All surgical procedures were successful and uneventful. The stromal layers of all the corneas were clear. Although mild epithelial edema could be observed in some cases by using a silt-lamp bio-microscopy at 20 min postoperatively but this sign disappeared at 24 h postoperatively. The main demographic and topographic data were shown in the Table 1.

**Table 1:** The main demographic and topographic data (*n* = 22)

Variables	Pre-operative		Postoperative 24 h		F value^a^	*P* value
	Mean ± SD	Range	Mean ± SD	Range		
Age (year)	28.4 ± 7.1	18 to 42	28.4 ± 7.1	18 to 42	–	–
MRSE (D)	−6.76 ± 1.39	−10.00 to −4.13	−0.22 ± 0.48	−1.38 to 0.75	508.580	<0.001^b^
K1 (D)	43.23 ± 1.40	40.1 to 45.2	38.35 ± 2.12	32.3 to 40.9	510.718	<0.001^b^
K2 (D)	44.56 ± 1.84	41.0 to 47.6	39.03 ± 2.23	32.7 to 42.3	557.309	<0.001^b^
Km (D)	43.88 ± 1.54	40.6 to 46.4	38.69 ± 2.15	32.5 to 41.6	622.613	<0.001^b^
Pentacam-CCT (μm)	546.6 ± 23.4	515 to 592	431.9 ± 28.0	399 to 501	521.421	<0.001^b^

*MRSE* manifest refraction spherical equivalent, *D* diopter, *K1* flat curvature power, *K2* flat curvature power, *Km* mean curvature power, *CCT*central corneal thickness

^a^Repeated measures analysis of variance (RM-ANOVA)

^b^Significant difference was detected

### IOP measurements

**Fig. 1:**
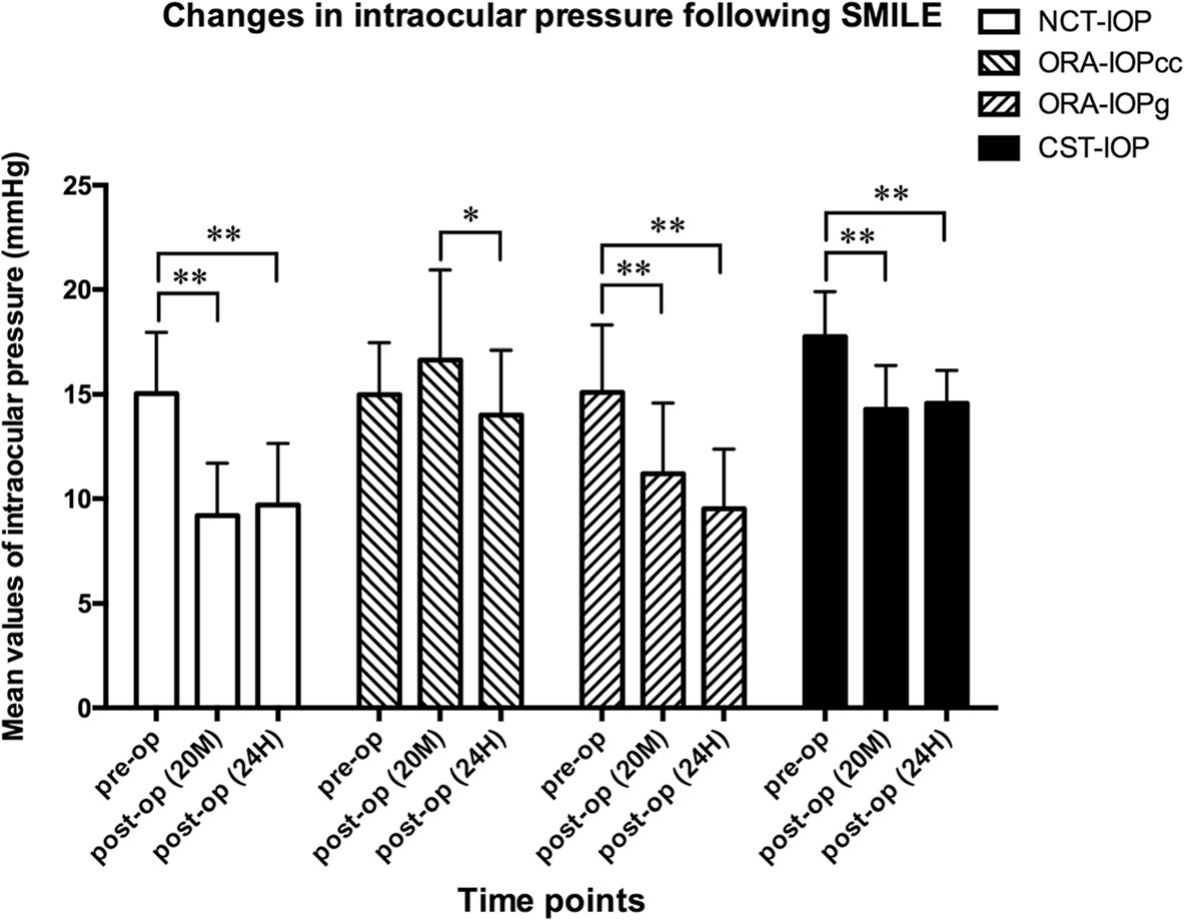
Changes in intraocular pressure following SMILE obtained by, NCT (NCT-IOP), ORA(ORA-IOPcc and ORA-IOPg) and CST (CST-IOP). Pre-op = postoperative; Post-op = postoperative; 20 M = 20 min; 24H = 24 h. “*” refers to Fisher’s least significant difference (LSD) post hoc *P* value <0.05; “**” refers to LSD post hoc *P* value < 0.01

As demonstrated in the Table 2, the measurements of NCT-IOP, IOPg, and CST-IOP significantly decreased after SMILE procedure (All *P* values <0.05). Fisher’s least significant difference (LSD) post hoc comparisons (Fig. 1) revealed that at 20 min postoperatively, the mean values of NCT-IOP (post hoc *P* < 0.001), IOPg (post hoc *P* < 0.001) and CST-IOP (post hoc *P* < 0.001) were all decreased dramatically when compared with the pre-operative values, but all of the values kept stable in the next 24 h (post hoc *P* = 0.365, post hoc *P* = 0.050 and post hoc *P* = 0.585, respectively). The mean values of IOPcc slightly increased at the 20-min mark (post hoc *P* = 0.056), but then deceased significantly at the 24-h mark (*P* = 0.028) however, no significant difference was detected between the IOPcc values obtained before and 24 h after SMILE (post hoc *P* = 0.051).

**Table 2:** Changes in IOP measurements (*n* = 22)

Variables	Pre-operative		Postoperative 20 min		Postoperative 24 h		F value^a^	*P* value
	Mean ± SD	Range	Mean ± SD	Range	Mean ± SD	Range		
NCT-IOP (mmHg)	15.04 ± 2.91	11.1 to 20.9	9.19 ± 2.52	4.6 to 15.2	9.70 ± 2.95	5.8 to 17.2	81.173	<0.001^b^
IOPcc (mmHg)	14.99 ± 2.46	10.2 to 19.0	16.65 ± 4.29	12.1 to 29.4	14.01 ± 3.10	6.8 to 20.3	4.975	0.030^b^
IOPg (mmHg)	15.10 ± 3.21	9.3 to 21.9	11.20 ± 3.38	4.7 to 18.2	9.52 ± 2.85	4.5 to 15.9	40.128	<0.001^b^
CST-IOP (mmHg)	17.74 ± 2.16	13.0 to 23.0	14.29 ± 2.09	10.0 to 17.5	14.57 ± 1.58	10.5 to 18.0	31.886	<0.001^b^

*IOPcc* corneal-compensated IOP, *IOPg* Goldmann-correlated IOP

^a^Repeated measures analysis of variance (RM-ANOVA)

^b^Significant difference was detected over time

### Changes in corneal biomechanical and deformation parameters

The definition of each deformation parameter was listed in the Table 3 [3, 13, 16]. As shown in the Table 4, the mean values of corneal hysteresis (CH), corneal resistance factor (CRF), AT1, AV1, AV2, Radius, DA all changed significantly after SMILE procedure (All *P* values <0.001). CH (post hoc *P* < 0.001), CRF (post hoc *P* < 0.001), AT1 (post hoc *P* < 0.001), AV1 (post hoc *P* = 0.007), AV2 (post hoc *P* = 0.016) and Radius (post hoc *P* = 0.001) decreased but DA increased remarkably (post hoc *P* < 0.001) at 20 min postoperatively. While the remaining parameters in the Table 4 all kept unchanged (All *P* values >0.05). Expect for the mean value of CH, which kept decreasing at 24 h postoperatively (post hoc *P* = 0.025), CRF, AT1, AV1, AV2, Radius and DA all remained stable at 24-h mark (All post hoc *P* values >0.05).

**Table 3:** Abbreviations for corneal deformation parameters

Abbreviation	Definition
AT1	Applination Time (time 1): the duration from the initiation to the moment that a cornea just deformed for the first time
ATh	Applination Time (hightest concavity): the duration from the initiation to the moment that a cornea is depressed to the highest concavity
AT2	Applination Time (time 2): the duration from the initiation to the moment that a corneal deformation just completely resumed
AL1	Applination length (time 1): the cord length recorded at the moment that a cornea just deformed for the first time
AL2	Applination length (time 2): the cord length recorded at the moment that a corneal deformation just completely resumed
AV1	Applination velocity (time 1): the instantaneous velocity recorded when a cornea just deformed
AV2	Applination velocity (time 2): the instantaneous velocity recorded when a cornea just completely resumed
Radius	Radius: the corneal radius obtained when a cornea is depressed to the highest concavity
PD	Peak Distance: the distance between the two corneal peaks recorded when a cornea is depressed to the highest concavity
DA	Deformation Amplitude: the maximum amplitude of corneal deformation recorded when a cornea is depressed to the highest concavity

**Table 4:** Changes in corneal biomechanical and deformation parameters (*n* = 22)

Variables	Pre-operative		Postoperative 20 min		Postoperative 24 h		F value^a^	*P* value
	Mean ± SD	Range	Mean ± SD	Range	Mean ± SD	Range		
CH (mmHg)	11.05 ± 1.81	8.7 to 16.7	6.82 ± 0.98	5.3 to 8.4	7.49 ± 1.32	5.6 to 11.0	90.283	<0.001^b^
CRF (mmHg)	10.84 ± 2.20	7.1 to 17.3	6.07 ± 1.26	2.9 to 9.3	6.09 ± 1.27	4.1 to 9.1	131.239	<0.001^b^
CST-CCT (μm)	545.8 ± 24.8	515.0 to 618.0	469.7 ± 46.4	389 to 580	440.9 ± 29.5	407 to 522	136.093	<0.001^b^
AT1 (ms)	7.28 ± 0.26	6.71 to 7.97	6.91 ± 0.23	6.52 to 7.32	6.90 ± 0.18	6.54 to 7.37	36.484	<0.001^b^
ATh (ms)	16.94 ± 0.53	16.17 to 17.79	16.77 ± 0.86	14.32 to 18.02	16.94 ± 0.63	15.71 to 18.02	0.465	0.631
AT2 (ms)	21.64 ± 1.29	16.89 to 22.82	22.13 ± 0.41	21.36 to 22.86	22.17 ± 0.29	21.61 to 22.87	3.042	0.089
AL1 (mm)	1.78 ± 0.04	1.71 to 1.87	1.72 ± 0.17	1.26 to 1.92	1.74 ± 0.20	1.19 to 1.94	0.798	0.429
AL2 (mm)	1.49 ± 0.39	0.44 to 2.00	1.35 ± 0.44	0.73 to 2.00	1.22 ± 0.41	0.73 to 1.89	2.281	0.131
AV1 (m/s)	0.16 ± 0.02	0.09 to 0.22	0.14 ± 0.02	0.08 to 0.19	0.15 ± 0.02	0.11 to 0.20	5.528	0.007^b^
AV2 (m/s)	−0.44 ± 0.14	−0.92 to −0.20	−0.56 ± 0.13	−0.75 to −0.33	−0.61 ± 0.16	−1.11 to −0.33	8.910	0.001^b^
PD (mm)	4.27 ± 1.19	2.36 to 5.43	4.51 ± 1.25	2.62 to 6.19	4.53 ± 1.27	2.57 to 6.23	0.355	0.704
Radius (mm)	6.97 ± 1.46	1.66 to 9.45	5.51 ± 1.00	1.78 to 6.88	5.69 ± 0.54	4.89 to 7.05	13.208	<0.001^b^
DA (mm)	1.09 ± 0.09	0.89 to 1.37	1.17 ± 0.10	1.03 to 1.43	1.14 ± 0.07	0.99 to 1.34	10.100	<0.001^b^

*MRSE* manifest refraction spherical equivalent, *D* diopter, *CH* corneal hysteresis, *CRF* corneal resistance factor, *CCT* central corneal thickness, *AT1* applanation time 1, *ATh* applanation time at the highest concavity; *AT2* applanation time 2, *AL1* applanation length 1; *AL2* applanation length 2, *AV1* applanation velocity 1, *AV2* applanation velocity 2, *PD* peak distance, *DA* deformation amplitude

^a^Repeated measures analysis of variance (RM-ANOVA)

^b^Significant difference was detected over time

### Correlations

Pearson’s correlation analysis showed that neither ΔNCT-IOP, ΔIOPcc, ΔIOPg nor ΔCST-IOP (“Δ” refers to the difference between the value obtained preoperatively and at 24 h postoperatively) significantly correlated with ΔCCT, ΔMRSE or ΔKm (all *P* values >0.05) But the post-operative CCT value measured with CST (CST-CCT) significantly correlated with CH (R = 0.511, *P* = 0.015) and CRF (R = 0.674, *P* = 0.001).

### Stepwise multiple linear regression models

The Table 5 demonstrated the statistically significant stepwise linear multiple regression models for predicting the theoretical postoperative values of NCT-IOP (Adjusted R^2^ = 0.400, F = 15.000, P = 0.001), IOPcc (Adjusted R^2^ = 0.472, F = 19.755, *P* < 0.001), IOPg (Adjusted R^2^ = 0.542, F = 25.850, *P* < 0.001) and CST-IOP (Adjusted R^2^ = 0.596, F = 11.336, *P* < 0.001).

**Table 5:** The stepwise multiple linear regression models for predicting theoretical post-op IOP values (*n* = 22)

Dependent Variables	Main Predictors	B^a^	SE^b^	t^c^	Sig.	β^d^	Regression Equation	Adjusted R^2^	F^e^	Sig.
Pre-op NCT-IOP	Practical postop NCT-IOP	0.646	0.167	3.873	<0.001	0.655	Theoretical post-op NCT-IOP (mmHg) = 0.646 × Practical post-op NCT-IOP(mmHg) + 8.774(mmHg)	0.400	15.000	<0.001
	Constant	8.774	1.688	5.198	0.001	-				
Pre-op ORAIOPcc	Practical postop IOPcc	0.558	0.126	4.445	<0.001	0.705	Theoretical post-op IOPcc (mmHg) = 0.558 × practical postoperative IOPcc (mmHg) + 7.166(mmHg)	0.472	19.755	<0.001
	Constant	7.166	1.800	3.981	0.001	-				
Pre-op ORAIOPg	Practical postopIOPg	0.848	0.167	5.084	<0.001	0.751	Theoretical post-op IOPg (mmHg) = 0.848 × practical postoperative IOPg (mmHg) + 7.024 (mmHg)	0.542	25.850	<0.001
	Constant	7.024	1.654	4.247	<0.001	-				
Pre-op CST-IOP	Post-op AT1	8.079	1.660	4.866	<0.001	0.680	Theoretical post-op CST-IOP (mmHg) = 8.079 × post-op AT1 (ms) + 0.035 × pre-op CCT(μm) + 0.515 × post-op PD (mm) - 59.47 mmHg	0.596	11.336	<0.001
	Pre-op CCT	0.035	0.013	2.732	0.014	0.379				
	Post-op PD	0.515	0.237	2.176	0.043	0.304				
	Constant	−59.47	13.524	−4.397	<0.001	-				

*Pre-op* pre-operative, *Post-op* post-operative, *AT1* applanation time 1, *CCT* central corneal thickness, *PD*peak distance, *Sig.* Significance

^a^Unstandardized Coefficients

^b^Standard Error of Unstandardized Coefficients

^c^Unstandardized Coefficients/Standard Error

^d^Standardized Coefficients (Beta)

^e^Multiple Linear Regression Model (Stepwise)

The practical post-operative NCT-IOP value was the main predictor of the theoretical post-operative NCT-IOP value (B = 0.646,t = 3.873,*P* = 0.001), but the theoretical post-operative NCT-IOP value did not associated with age, preoperative CCT, postoperative CCT, corneal curvature or ΔMRSE (All *P* values > 0.05).

For IOPcc and IOPg, the practical post-operative IOPcc and IOPg values were the main predictors (B = 0.558, t = 4.445, *P* < 0.001; B = 0.848, t = 5.084, *P* < 0.001, respectively) for predicting theoretical post-operative IOPcc and IOPg value. But both the theoretical post-operative IOPcc and IOPg values did not associated with age, preoperative CCT, postoperative CCT, postoperative CH, postoperative CRF, corneal curvature or ΔMRSE (All *P* values > 0.05).

For CST-IOP, the postoperative AT1 value (B = 8.079, t = 4.866, *P* < 0.001), preoperative CCT value (B = 0.035, t = 2.732, *P* = 0.014) and postoperative PD value (B = 0.515, t = 2.176, *P* = 0.043) were the main predictors of the theoretical post-operative CST-IOP value. But the theoretical post-operative CST-IOP value did not associated with age, postoperative CCT, corneal curvature, ΔMRSE or other postoperative CST parameters (All *P* values >0.05).

## Discussion

Corneal refractive surgeries compromise corneal mechanical strength, thus leading to underestimated IOP, which may obscure the diagnosis of steroid-induced glaucoma or open-angle glaucoma during postoperative follow-ups [17, 18].

In the present study, the mean values of NCT-IOP, IOPcc, IOPg and CST-IOP significantly deceased at 24 h postoperatively. Post-operative CCT significantly correlated with CH and CRF, but ΔCCT, as well as ΔMRSE and ΔKm, did not correlated with ΔNCT-IOP, ΔIOPcc, ΔIOPg or ΔCST-IOP, indicating that ΔCCT, ΔMRSE and corneal curvature may not be the main factors affecting IOP measurements. CH and CRF reflect the characters of force absorption and the resistance of the cornea, respectively. They are both dependent to corneal thickness [19]. Vestergaard AH, et al. [20] reported CH and CRF still significantly correlated with CCT after SMILE procedure, while Shah S [15] reported that ΔCH and ΔCRF did not correlated with ΔCCT after corneal refractive surgeries. A possible explanation is that comparing with the removed corneal tissue, the residual cornea contributed much more effect in maintaining the entire corneal biomechanical strength.

The stepwise multiple linear regression model analysis revealed that the practical post-operative IOP values obtained using NCT and ORA were the main predictors of the theoretical post-operative IOP values (Pre-operative IOP values). The models explained 40.0, 47.2 and 54.2% of the variance assessed using NCT-IOP, IOPcc and IOPg respectively. For CST, the postoperative AT1 value, preoperative CCT value and postoperative PD value were the main predictors of the theoretical post-operative CST-IOP value. CST-IOP model explained 59.6% of the variance.

We found it is interesting that the change in CCT seems to be independent of the change in IOP value after SMILE procedure as neither ΔCCT value was correlated with ΔNCT-IOP, ΔIOPcc, ΔIOPg or ΔCST-IOP nor CCT was a predictor of theoretical post-operative IOP value. So far, various instruments including NCT [21], ORA [22], CST, Goldmann applanation tonometry (GAT) and dynamic contour tonometry (DCT) [23] have been employed to investigate the changes in IOP values after LASIK. Cheng AC, et al. [21] reported the preoperative NCT-IOP significantly correlated with the postoperative one after LASIK. Moreover, postoperative CCT and ablation depth were included into their model, which was different from ours. Chen S and his colleagues [22] studied the changes in ORA parameters after myopic LASIK. They found that ΔIOPg was positively correlated with ΔCCT, but ΔIOPcc was not. In addition, they found ablation depth was correlated with ΔCRF and ΔCH. It is also reported that CST and GAT would underestimate IOP following LASIK procedure [24]. The underlying reasons for these discrepancies may be the difference in surgical techniques and their efforts on corneal biomechanical structure. During LASIK procedure, stromal flap creation cuts almost all the collagen fibers in the anterior stroma of a cornea and compromised the integrality of the Bowman’s layer, moreover, excimer laser ablated the stromal tissue, which contributes most of the biomechanics of the entire cornea. So the residual stromal bed undertakes the main role to maintain the corneal shape and biomechanical stability, in addition, the more corneal tissue removed, the lower the postoperative IOP value will be. However, SMILE procedure neither creates a stromal flap nor ablates Bowman’s layer and the anterior stroma. As the corneal collagen fibers in the anterior stromal layer is much compact than that in the middle or posterior layer [25], most of the corneal biomechanical properties are remained. Reinstein DZ, et al. [14] developed a mathematical model to estimate the postoperative stromal tensile strength following SMILE, PRK and LASIK. They found that SMILE lenticule thickness could be approximately 100 μm greater than the LASIK ablation depth, which is equivalent to approximately 7.75 diopters, and still have equivalent corneal strength. Wang D, et al. [26] found an interesting phenomenon that in myopia of −6.00D or less, the ΔCH and the ΔCRF value between SMILE and LASIK were not significant. But in myopia greater than −6.00D, CH and CRF decreased more in LASIK than in SMILE. Another reason is that the NCT, ORA and CST may not be sensitive enough to detect the linear correlations between ΔIOP and ΔCCT as the residual cornea following SMILE contributes much more biochemical stability than the removed leuticule. We noticed that although the IOPcc values obtained at 20-min mark increased slightly but returned to pre-operative level at 24 h postoperatively, indicating corneal suction and surgical operation might also affect the postoperative IOP assessment [27]. As IOPcc is adjusted to account for CH, which is significantly correlated with CCT value, the IOP gap can be compensated [28, 29]. Osman IM [16] reported similar findings, indicating that practical post-operative IOPcc value may be used to assess IOP after SMILE procedure.

The limitations of the present study are as follows. As the Goldmann applanation tonometer (GAT) IOP measurement has potential risk of infection, additionally, fluorescein sodium eye drops may infiltrate into the interlayer when GAT is employed for IOP assessment during the early post-operative phase, we were not able to obtain the GAT-IOP values. But the purpose of the study is to investigate the changes in IOP before and after SMILE with the same tonometer rather than to investigate the interrelations among the IOP values obtain with different instruments, GAT is not indispensable in the present study. As the biomechanical properties of the corneas underwent SMILE procedure may fluctuate during long-term follow-up, further studies are required to validate if the models were still effective in long-term outcomes of SMILE.

## Conclusions

IOP values were underestimated when assessed by using NCT-IOP, IOPg and CST-IOPg after SMILE procedure. Both practical postoperative IOPcc values and theoretical post-operative CST-IOP values may be more preferable for IOP assessment or management following SMILE procedure.

## Abbreviations

AL1: Applination length (time 1) is the cord length recorded at the moment that a cornea just deformed for the first time
AL2: Applination length (time 2) is the cord length recorded at the moment that a corneal deformation just completely resumed
AT1: Applination Time (time 1) is the duration from the initiation to the moment that a cornea just deformed for the first time
AT2: Applination Time (time 2) is the duration from the initiation to the moment that a corneal deformation just completely resumed
ATh: Applination Time (hightest concavity) is the duration from the initiation to the moment that a cornea is depressed to the highest concavity
AV1: Applination velocity (time 1) is the instantaneous velocity recorded when a cornea just deformed
AV2: Applination velocity (time 2): the instantaneous velocity recorded when a cornea just completely resumed
DA: Deformation Amplitude is the maximum amplitude of corneal deformation recorded when a cornea is depressed to the highest concavity
PD: Peak distance is the distance between the two corneal peaks recorded when a cornea is depressed to the highest concavity
Radius: Is the corneal radius obtained when a cornea is depressed to the highest concavity
